# The Saturne cementless dual-mobility cup grants satisfactory long-term survival

**DOI:** 10.1186/s40634-022-00542-3

**Published:** 2022-10-11

**Authors:** Thierry Gaillard, Sonia Ramos-Pascual, Mo Saffarini, Jean-Pierre Piton

**Affiliations:** 1 Polyclinique du Beaujolais, 120 Anc. Rte de Beaujeu, 69400 Arnas, France; 2 ReSurg SA, Rue Saint-Jean 22, 1260 Nyon, Switzerland; 3 UNEOS - Hôpitaux Privés de Metz, 15 Rue de Sarre, 57070 Metz, France

**Keywords:** Total hip arthroplasty, Long-term outcomes, Clinical scores

## Abstract

**Purpose:**

To report long-term survival and clinical outcomes of primary total hip arthroplasty (THA) using a Saturne cementless dual-mobility (DM) cup, and investigate whether patient demographics or surgical parameters affect clinical scores.

**Methods:**

A consecutive series of primary THAs implanted with Saturne cementless DM cups between 01/09/2009–31/12/ 2011 was retrospectively assessed. Patients were postoperatively evaluated using modified Harris hip score (mHHS) and forgotten joint score (FJS). Complications, reoperations, and revisions were noted. Regression analyses were performed to determine associations of postoperative mHHS with preoperative and intraoperative variables. Ten-year Kaplan–Meier survival was calculated.

**Results:**

Of 308 patients (308 hips), 111 (36%) had died with their original cups in place, 29 (9%) were lost-to-follow-up, and 5 (2%) required cup revision, leaving a final cohort of 163 (53%) with their original cup in place at a follow-up of ≥ 10 years. Ten-year survival was 98% considering cup revision for any reason as endpoint; 99% considering cup revision for aseptic loosening as endpoint; 96% considering stem revision for any reason as endpoint; and 96% considering any revision as endpoint. The final cohort of 163 patients was assessed at 11 ± 1 years (range, 10–13), mHHS was 85 ± 16 (range, 31–100) and FJS was 84 ± 24 (range, 0–100). Multivariable regression analysis revealed that postoperative mHHS significantly worsened with age (β = -0.48, *p* = 0.007) and BMI (β = -0.70, *p* = 0.008), as well as for 22 mm head sizes (β = -6.98, *p* = 0.046).

**Conclusions:**

The Saturne DM cup granted satisfactory survival and clinical outcomes at a minimum follow-up of 10 years, and resulted in no cases of intra- or extra-prosthetic dislocations.

## Introduction

The use of dual-mobility (DM) cups in total hip arthroplasty (THA) has gained popularity over the last few decades, as their advantageous head-to-neck ratio and double articulation result in lower dislocation risk and greater range of motion [5, 16]. First- and second- generation DM cups were associated with cases of intra-prosthetic dislocation and aseptic loosening; however, modern DM cups have shown low revision rates and good clinical outcomes, even in unselected populations [1, 7, 22].

In 2000, the Saturne cementless DM cup (Amplitude, France) was introduced with a quasi-anatomic rim to prevent dislocations without increasing risks of psoas impingement. The rim is therefore augmented at the postero-superior half to increase the ‘jump distance’ but follows the hemispherical equator at the antero-inferior half to avoid prosthetic overhang at the psoas valley [17, 18]. In a recent study, Gaillard et al. [8] reported the 10-year survival and clinical outcomes of 310 THAs using the Saturne DM cup implanted by one surgeon at a university hospital using a posterolateral approach. These encouraging findings have not yet been corroborated by other surgeons at private hospitals, who may use different surgical techniques and implant combinations.

The purpose of this study was to confirm previous findings by reporting long-term survival and clinical outcomes of primary THA using a Saturne cementless DM cup, and to investigate whether patient demographics or surgical parameters affect clinical scores.

## Materials and methods

### Study design

The authors retrospectively assessed a consecutive series of patients that underwent primary THA between September 2009 and December 2011 by 2 surgeons at 2 centers. Patients were included in the present study if they received the Saturne cementless DM cup. Patients were excluded from the study if they did not have ≥ 10 years follow-up. The initial cohort comprised 308 patients (308 hips), of which 150 were males and 158 were females, aged 74 ± 9 years (range, 42–91) at index surgery with body mass index (BMI) of 28 ± 5 kg/m^2^ (range, 18–52) (Table 1). Indications for surgery were primary osteoarthritis (*n* = 270, 87.7%), secondary osteoarthritis (*n* = 12, 3.9%), femoral neck fracture (*n* = 12, 3.9%), avascular necrosis (*n* = 11, 3.6%), and rheumatoid arthritis (*n* = 3, 1.0%). All patients gave informed consent to participate in the study, and this study was approved by the institutional review board of GCS Ramsay Santé pour l’Enseignement et la Recherche (IRB number: COS-RGDS-2022-08-001-GAILLARD-T).

**Table 1:** Pre- and intra-operative data

	**Original Cohort**	
	(*n* = 308 hips)	
	Mean ± SD or n (%)	Range
**Preoperative data**		
Age	73.6 ± 8.8	(42- 91)
BMI	27.8 ± 4.6	(18- 52)
HHS	34.1 ± 10.4	(6- 66)
Male sex	150 (49%)	
Indication		
Primary OA	270 (88%)	
Secondary OA	12 (4%)	
Femoral neck fracture	12 (4%)	
Avascular necrosis	11 (4%)	
RA	3 (1%)	
**Intraoperative data**		
Surgical approach		
Anterolateral	300 (97%)	
Anterior	6 (2%)	
Posterolateral	2 (1%)	
Navigation		
No	196 (64%)	
Yes	112 (36%)	
Stem type		
Modular Acor	108 (35%)	
Fixed neck Integrale	166 (54%)	
Modular Integrale	34 (11%)	
Stem fixation		
Cemented	49 (16%)	
Cementless	259 (84%)	
Neck length		
Short	46 (15%)	
Medium	207 (67%)	
Long	55 (18%)	
Head size		
22	136 (44%)	
28	172 (56%)	
Cup size		
44	1 (0%)	
46	7 (2%)	
48	53 (17%)	
50	53 (17%)	
52	55 (18%)	
54	61 (20%)	
56	56 (18%)	
58	16 (5%)	
60	6 (2%)	

Abbreviations: *BMI* body mass index, *HHS* Harris hip score, *OA* osteoarthritis, *RA* rheumatoid arthritis, *SD* standard deviation

### Surgical information

The surgical approach was anterolateral in most cases (*n* = 300, 97%), while it was anterior (*n* = 6, 2%) or posterolateral (*n* = 2, 1%) in few cases (Table 1). Navigation with a computer assisted surgery system (Amplivision, Amplitude, France) was used in over one-third of cases (*n* = 112, 36%). Three types of stems were used: the Integrale cementless fixed-neck stem (*n* = 116, 54%) (Amplitude, France), the Integrale cementless modular stem (*n* = 34, 11%) (Amplitude, France), and the Acor modular stem (*n* = 108, 35%) (Amplitude, France) available cementless and cemented.

### Clinical assessment

Patients were evaluated preoperatively at the clinic using the Harris hip score (HHS). The latest clinical evaluation was performed via telephone by an independent observer, who recorded the modified HHS (mHHS), forgotten joint score (FJS), and satisfaction level (very satisfied, satisfied, disappointed, dissatisfied). Complications, reoperations, and revisions were noted from patient files and from phone interviews with patients. If a patient was deceased, the family doctor or next of kin was contacted for the date of death and to confirm that the patient died with the cup in place.

### Statistical analyses

Descriptive statistics were used to summarise patient demographics, surgical data, and clinical outcomes. Preoperative HHS was converted into mHHS to calculate the net change in score. Univariable linear regression analyses were performed to determine associations of postoperative mHHS with 9 variables (age, BMI, sex, use of navigation, stem type, stem fixation, neck length, head size, and cup size). The variables: indication, surgical approach, and cup size were not included in regression analyses because at least one subgroup had less than 10 patients. The variables: stem type, stem fixation, and navigation were not included in multivariable regression analyses because of significant collinearities between them. Furthermore, the Kaplan–Meier (KM) method was used to estimate survival and 95% confidence intervals (CI) at 10 years for 4 different end points: (i) cup revision for any reason; (ii) cup revision for aseptic reasons; (iii) stem revision for any reason; (iv) any revision for any reason. Statistical analyses were performed using R version 4.1.3 (R Foundation for Statistical Computing, Vienna, Austria). *P*-values < 0.05 were considered statistically significant.

## Results

### Complications, reoperations, revisions, deaths, and losses to follow-up

From the initial cohort of 308 patients (308 hips), two (0.6%) patients had complications that did not require reoperation: one (0.32%) had an infected hematoma treated with antibiotics and one (0.32%) had a femoral fracture for which revision was contraindicated because the patient was too frail for surgery. It is important to note that there were no intra- or extra-prosthetic dislocations. Six (1.9%) patients required reoperations without implant removal: two (0.65%) had lavage/debridement for infection and four (1.3%) had osteosynthesis using plate and screws for femoral fracture. Four (1.3%) patients required cup and stem revision: two (0.65%) for periprothstic joint infection at 1 and 3 years, one (0.32%) for aseptic loosening at 6 years, and 1 (0.32%) for femoral fracture at 7 years. One (0.3%) patient required cup revision only, for psoas impingement at 8 years. Six (1.9%) patients required stem revision only: five (1.6%) for femoral fracture at 0, 1, 3, 8 and 10 years, and one (0.32%) for fracture of the modular stem neck at 4 years.

**Fig. 1:**
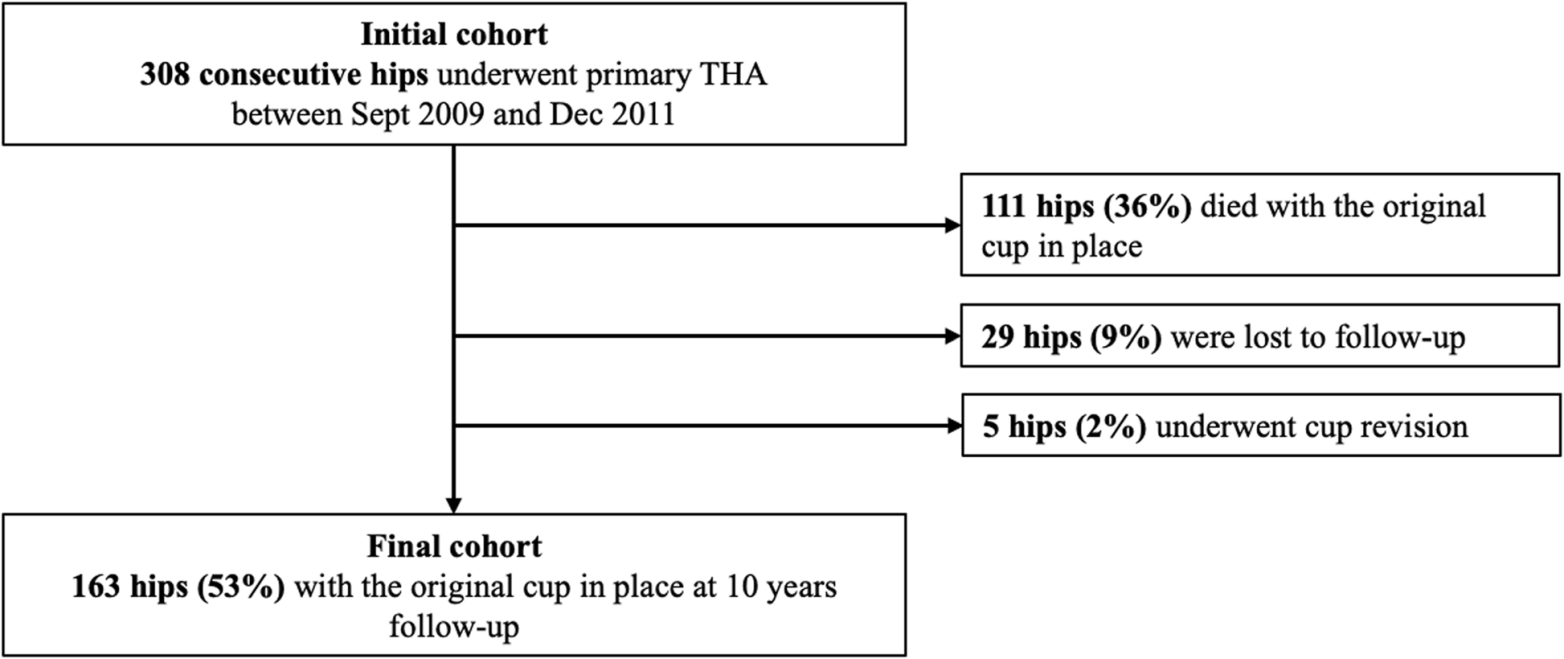
Flowchart indicating that from the initial cohort of 308 patients (308 hips), 111 (36%) had died with their original cups in place, 29 (9%) were lost to follow-up, and five (1.6%) required cup revision, thus leaving a final cohort of 163 patients (53%) with their original cup in place with a follow-up of ≥ 10 years

At a minimum follow-up of 10 years, 111 (36%) patients had died with their original cups in place and 29 (9%) could not be reached, but their most recent follow-up records indicated that none had cup revision (Fig. 1). Patients were excluded from the final cohort if they had died, were lost to follow-up or had cup revision; thus, the final cohort comprised 163 patients (53%) with their original cup in place with a follow-up of ≥ 10 years.

### Survival

**Fig. 2:**
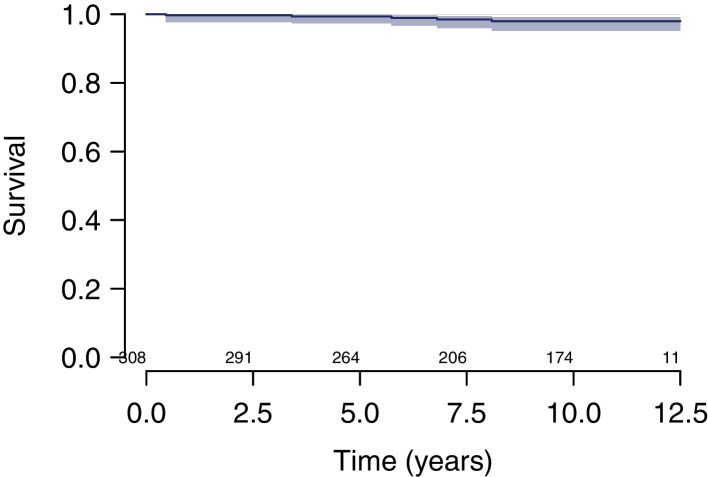
Survival at 10 years estimated using the KM method was 98.0% (CI, 95.1–99.2%) considering cup revision for any reason as endpoint

**Fig. 3:**
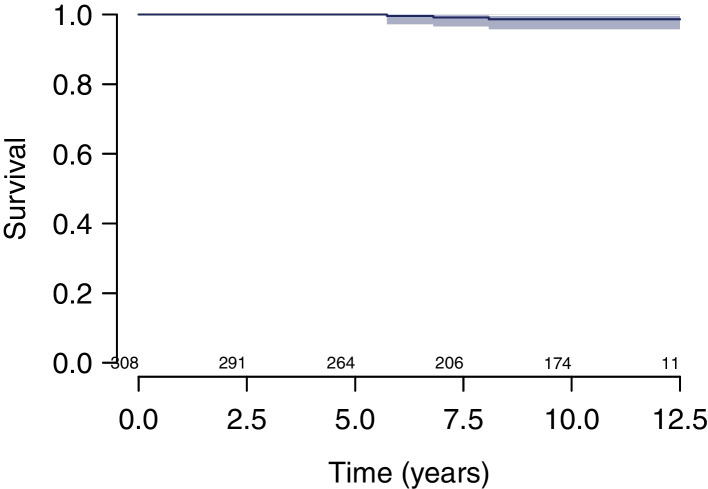
Survival at 10 years estimated using the KM method was 98.6% (CI, 95.8–99.6%) considering cup revision for aseptic loosening as endpoint

**Fig. 4:**
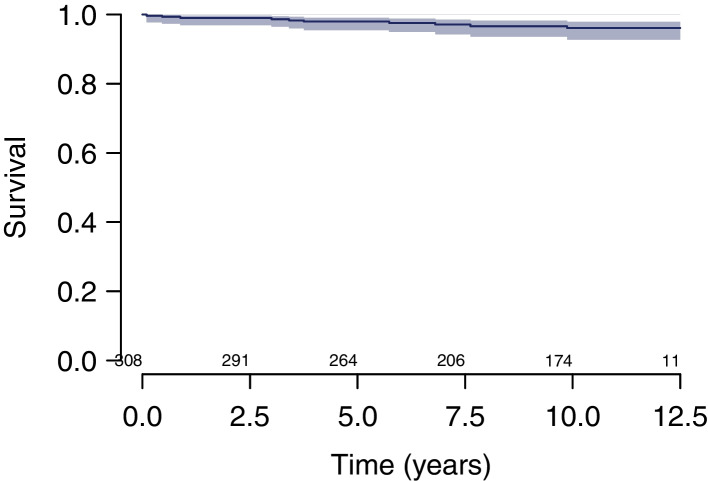
Survival at 10 years estimated using the KM method was 96.1% (CI, 92.8–97.9%) considering stem revision for any reason as endpoint

**Fig. 5:**
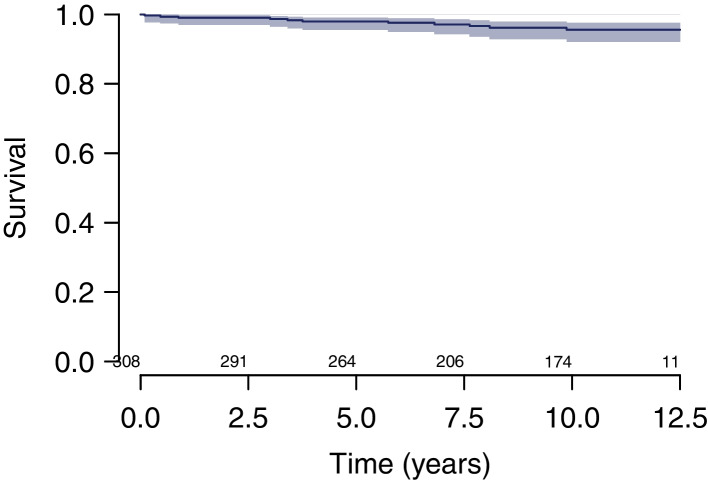
Survival at 10 years estimated using the KM method was 95.6% (CI, 92.1–97.6%) considering any revision for any reason as endpoint

Survival at 10 years estimated using the KM method was (i) 98.0% (CI, 95.1–99.2%) considering cup revision for any reason as endpoint (Fig. 2); (ii) 98.6% (CI, 95.8–99.6%) considering cup revision for aseptic loosening as endpoint (Fig. 3); (iii) 96.1% (CI, 92.8–97.9%) considering stem revision for any reason as endpoint (Fig. 4); and (iv) 95.6% (CI, 92.1–97.6%) considering any revision for any reason as endpoint (Fig. 5).

### Clinical outcomes

The final cohort of 163 patients (163 hips) comprised 80 males and 83 females, aged 82 ± 7 years (range, 64–88) at a follow-up of 11 ± 1 years (range, 10–13). The postoperative mHHS was 85 ± 16 (range, 31–100) and the net change in mHHS was 53 ± 20 (range, -19–82; *p* < 0.001) (Table 2). The postoperative FJS was 84 ± 24 (range, 0–100). Most patients were very satisfied (*n* = 104, 64%) or satisfied (*n* = 53, 33%) with surgery.

**Table 2:** Clinical scores and satisfaction with surgery

	Cohort with ≥ 10 year FU	
	(*n* = 163 hips)	
	Mean ± SD or n (%)	Range
**Follow-up ***(years)*	11.3 ± 0.7	(10.0 – 12.6)
**Modified HHS**		
*Preoperative*	32.2 ± 10.8	(3 – 55)
*Postoperative*	85.4 ± 16.3	(31 – 100)
*Net change*	53.2 ± 20.4	(-19 – 82)
**Postoperative FJS**	84.0 ± 23.6	(0 – 100)
**Satisfaction with surgery**		
*Very satisfied*	104 (64%)	
*Satisfied*	53 (33%)	
*Dissapointed*	4 (2%)	
*Dissatisfied*	2 (1%)	

Abbrevations: *HHS* Harris hip score, *FJS* forgotten joint score, *FU* follow-up, *SD* standard deviation

Univariable linear regression analyses revealed that postoperative mHHS significantly worsened with BMI (β = -0.68, *p* = 0.011), as well as for 22 mm head sizes (β = -6.38, *p* = 0.012), while it was significantly better for navigated surgery (β = 10.55, *p* < 0.001), modular Acor stems (β = 11.24, *p* < 0.001), short neck stems (β = 10.68, *p* = 0.003), and long neck stems (β = 8.56, *p* = 0.012) (Table 3). Multivariable linear regression analysis confirmed that postoperative mHHS significantly worsened with age (β = -0.48, *p* = 0.007) and BMI (β = -0.70, *p* = 0.008), as well as for 22 mm head sizes (β = -6.98, *p* = 0.046).

**Table 3:** Uni- and multi-variable regression analyses of postoperative modified Harris hip score

		**Univariable**			**Multivariable**		
	**n**	**β**	**95%CI**	***P*-value**	**β**	**95% C.I**	***P*-value**
**Preoperative data**							
Age	163	-0.29	(-0.64 – 0.06)	0.099	-0.48	(-0.83 – -0.13)	**0.007**
BMI	163	-0.68	(-1.21 – -0.16)	**0.011**	-0.70	(-1.21 – -0.19)	**0.008**
Male sex	80	2.78	(-2.26 – 7.82)	0.278	0.12	(-6.41 – 6.64)	0.972
**Intraoperative data**							
Navigation							
No	113	REF					
Yes	50	10.55	(5.29 – 15.81)	** < 0.001**			
Stem type							
Modular Acor	47	11.24	(5.75 – 16.73)	** < 0.001**			
Fixed neck Integrale	99	REF					
Modular Integrale	17	1.62	(-6.45 – 9.69)	0.693			
Stem fixation							
Cemented	17	1.16	(-7.09 – 9.42)	0.781			
Cementless	146	REF					
Neck length							
Short	25	10.68	(3.81 – 17.56)	**0.003**	6.46	(-1.55 – 14.47)	0.113
Medium	111	REF					
Long	27	8.56	(1.89 – 15.22)	**0.012**	6.62	(-0.15 – 13.40)	0.055
Head size							
28	86	REF					
22	77	-6.38	(-11.35 – -1.41)	**0.012**	-6.98	(-13.82 – -0.13)	**0.046**
Cup size							
Small (44–48)	36	-6.06	(-12.39 – 0.28)	0.061	-3.60	(-10.50 – 3.31)	0.305
Medium (50–54)	88	REF					
Large (56–60)	39	-1.66	(-7.82 – 4.50)	0.595	-5.52	(-13.08 – 2.04)	0.151

Abbreviations: *BMI* body mass index, *REF* reference, *CI* confidence interval

## Discussion

The principal findings of this study are that, even when implanted in various settings and with different implant combinations, the Saturne DM cup confirmed satisfactory survival and clinical outcomes at a minimum follow-up of 10 years. The Saturne DM cup was introduced in 2000, with a quasi-anatomic rim to prevent dislocations without increasing risks of psoas impingement, augmented at the postero-superior half to increase the ‘jump distance’ but following the hemispherical equator at the antero-inferior half to avoid prosthetic overhang at the psoas valley [17, 18]. Only one previous study has reported on 10-year survival and clinical outcomes of this DM cup, in a series of 300 THAs performed by one surgeon using a posterolateral approach at a university hospital, but these findings had not been corroborated in other settings. In the present series, there were no cases of intra- or extra-prosthetic dislocations, indicating that the quasi-anatomic rim is effective at increasing the jump distance without causing neck-cup impingement. The secondary findings of this study are that, in multi-variable linear regression analysis, postoperative mHHS was significantly worse for 22 mm head sizes, as well as for patients of greater age and BMI.

Modern cementless DM cups are designed with a thin metal shell, and have porous coating on their outer surface to favour bone ingrowth, and are mirror-polished on their inner surface to optimise articulation against a large-diameter polyethylene (PE) insert, which assembles with a small retentive femoral head. The present study included young patients (< 55 years), obese patients (BMI > 30 kg/m^2^), as well as patients with femoral neck fracture, who may have higher risks of dislocations; nonetheless, according to the systematic review by Batailler et al. [1], DM cups provide satisfactory outcomes, which are better than those provided by standard cups, in these populations. The findings of the present study are in line with those of other modern DM cups. Ten-year survival of the acetabular cup with revision for any reason as endpoint in the literature ranged between 95–100% [7, 8, 13, 15], which is comparable to the present study (98.0%). Intra- and extra-prosthetic dislocation rates in the literature ranged between 0–2.4% and 0–1.9% respectively [2, 3, 7, 8, 13, 21], with a recent systematic review stating that there are no intraprosthetic dislocations with modern DM cups [1], these are comparable to the intra- and extra-prosthetic dislocation rates of the present study (0%). Finally, the average HHS in the literature ranged between 84–95 [2, 3, 8, 13, 21], which is comparable to the mHHS of the present study (85 ± 16).

The present series had a revision rate of 1.6%, with deep infection being the main cause of revision (0.6%), followed by aseptic loosening (0.3%), femoral fracture (0.3%), and psoas impingement (0.3%). The present revision rate is comparable to that reported for other modern DM cups; while studies on smaller series (40–104 hips) had no revisions at 5–10 years of follow-up [12, 14, 19, 20], larger cohorts (167–3474 hips) had revision rates of 0.5–3.6%, at 5–13 years of follow-up [3, 4, 6–9, 11].

The study by Gaillard et al. [8] evaluated 310 THAs using the Saturne cementless DM cup and the Integrale cementless stem with head sizes of 22 mm (36% of hips) or 28 mm (64% of hips), implanted using a posterolateral approach by one surgeon at a university hospital. The study reported 10-year survival considering any revision as endpoint of 98% (CI, 97–99%), postoperative HHS of 95 (range, 76–100), and no intra- or extra-prosthetic dislocations. In contrast, the present study used three different types of stems (Integrale cementless fixed-neck stems in 54% of hips, Integrale cementless modular stems in 11% of hips, and Acor cementless/cemented modular stems in 35% of hips) with head sizes of 22 mm (44% of hips) or 28 mm (56% of hips) implanted using an anterolateral approach in most cases (97% of hips) by two surgeons at private hospitals. The present study reported a comparable 10-year survival considering any revision as endpoint of 96% (CI, 92–98%), a lower postoperative mHHS of 85 (range, 31–100), and also no intra- or extra-prosthetic dislocations. It is worth noting that Gaillard et al. [8] had a smaller proportion of 22 mm heads (36% vs 44%), and included patients of younger age (68 [range, 40–84] vs 74 [range, 42–91]) and lower BMI (25 [range, 18–43] vs 28 [range, 18–52]).

The effect of patient demographics and intraoperative parameters on clinical outcomes were evaluated using multivariable linear regression analysis, which revealed significantly worse postoperative mHHS for 22 mm head sizes (β = -6.98, *p* = 0.046), as well as for patients of greater age (β = -0.48, *p* = 0.007) and BMI (β = -0.70, *p* = 0.008). It is important to note that use of navigation, stem type, and stem fixation were not included in multivariable analysis because of significant collinearities between them. In a previous large series, Fessy et al. [7] advised against the use of 22 mm heads, as they are associated with increased risks of intraprosthetic dislocations due to reduced neck-to-head ratio, favoring earlier impingement between the neck of the stem and the retaining ring of the cup. While there were no intraprosthetic dislocations in the present series, it is possible that 22 mm heads could have limited range of motion, and thereby compromise mHHS in some patients [7, 10, 23].

This retrospective study has a number of limitations. First, radiographs were not available, and therefore the effect of navigated versus manual surgery on cup positioning could not be investigated. Second, the net change in HHS could not be calculated, because postoperatively an independent observer assessed mHHS via telephone; patients were not asked to go to the clinic routinely to reduce the risk of exposure to COVID-19. Instead, the net change in mHHS is presented. Third, not all intraoperative data could be included in regression analyses as some variables had subgroups with less than 10 hips and other variables demonstrated significant collinearities.

## Conclusions

This study has demonstrated that, even when implanted in various settings and with different implant combinations, the Saturne DM cup granted satisfactory survival and clinical outcomes at a minimum follow-up of 10 years, and resulted in no cases of intra- or extra-prosthetic dislocations.

## Data Availability

The datasets used and/or analysed during the current study are available from the corresponding author on reasonable request.
